# Supplementary material for: Anti-Spike Antibody Response to Natural Infection with SARS-CoV-2 and Its Activity against Emerging Variants
Source: Microbiol Spectr. 2022 Jun 15;10(4):e00743-22. doi: 10.1128/spectrum.00743-22 (PMC9430469; doi:10.1128/spectrum.00743-22)
Supplement: Supplemental file 1 — Tables S1 and S2; Fig. S1-S4. Download spectrum.00743-22-s0001.pdf, PDF file, 0.2 MB [file spectrum.00743-22-s0001.pdf]

Anti-spike antibody response to natural infection with SARS-CoV-2 and its activity against emerging variants

1. Table S1. Demographic and clinical manifestations of 25 COVID-19 patients.
2. Table S2. Serological anti-Spike antibody response of 25 COVID-19 patients.
3. Fig. S1. Relationship of serological spike-binding, RBD-binding, hemagglutination and ACE2-blocking activity among COVID-19 patients.
4. Fig. S2. (A) Relationship of fever duration and peak viral load among COVID-19 patients. (B) Relationship of fever duration and RBD-binding, hemagglutination and ACE2-blocking activity among COVID-19 patients.
5. Fig. S3. Analysis of spike-binding antibody response and hemagglutination serological titer at enrolment and follow-up.
6. Fig. S4. Relationship of serological spike or RBD-binding activity and neutralizing activity of convalescent sera.

Table S1. Demographic and clinical manifestations of 25 COVID-19 patients.

Patient	Gender	Age	Symptoms and signs	Fever duration (Day)	Clinical diagnosis	Convalescent samples (Day after onset)	Follow-up samples (Month after onset)
1	F	55	Fever, cough	12	Pneumonia	D15, 19, 23, 27	M15
2	M	52	Fever	5	Febrile illness	D6, 10, 14, 18	n/a
3	M	30	Cough	0	URI	D13	n/a
4	F	26	Fever, cough	3	URI	D10	n/a
5	F	23	Cough	0	URI	D9	n/a
6	M	43	Fever, cough	7	Pneumonia	D5, 11, 13, 21	M13
7	F	28	Fever	4	Febrile illness	D11	M13
8	F	22	Rhinorrhea	0	URI	D18	n/a
9	F	21	Fever, rhinorrhea	2	URI	D6	n/a
10	F	63	Cough	0	URI	D9, 17	n/a
11	M	41	Diarrhea	0	Viral syndrome	D7, 16	n/a
12	M	32	Fever, cough	1	URI	D6, 14	n/a
13	F	43	Cough	0	URI	D33	n/a
14	M	31	Fever, cough	8	Pneumonia	D25	M7
15	M	43	Cough	0	URI	D22, 26	n/a
16	F	22	Sorethroat	0	URI	D29, 33	n/a
17	F	36	Fever, cough	4	URI	D21	n/a
18	M	41	Sorethroat	0	URI	D29, 33	n/a
19	M	20	Fever, sorethroat	4	URI	D21, 25, 33	M11
20	M	58	Fever, cough	0	URI	D26	n/a
21	M	46	Fever, cough	3	URI	D24	M11
22	M	55	Fever, cough	1	URI	D11, 15, 19	n/a
23	F	56	Fever, cough	10	Pneumonia	D24, 28	M9
24	F	28	Nausea	0	Viral syndrome	D18, 30	n/a
25	M	52	Fever, cough	12	Pneumonia	D23, 30, 39	M8

Abbreviations: F, female; M, male; URI, upper respiratory tract infection; D, day; M, month; n/a, not available.

Table S2. Serological anti-Spike antibody response of 25 COVID-19 patients.

Patient	Time after onset	Anti-Spike binding activity (%)	Anti-RBD binding activity (%)	ACE2-blocking activity (reciprocal titer)*	Hemagglutination activity (reciprocal titer)#	Spike-specific IgG/IgM/IgA memory B cell frequency (%)$
1	D15	45.20	8.12	Neg	40	n/a
	D19	57.30	54.04	n/a	320	n/a
	D23	68.60	78.84	n/a	n/a	n/a
	D27	64.20	77.20	3.37	640	1.03/1.69/0.14
	M15	21.20	n/a	n/a	Neg	n/a
2	D6	42.60	6.14	Neg	320	n/a
	D10	35.40	28.14	n/a	320	n/a
	D14	57.70	37.36	n/a	320	n/a
	D18	54.40	43.86	Neg	320	0.63/1.36/0.22
3	D13	4.40	2.18	Neg	Neg	0.00/0.00/0.00
4	D10	9.00	1.80	Neg	20	0.00/0.48/0.00
5	D9	3.70	1.30	Neg	Neg	0.00/0.00/0.00
6	D5	5.00	1.68	Neg	Neg	n/a
	D11	62.50	39.54	n/a	320	n/a
	D13	60.60	68.78	n/a	n/a	n/a
	D21	68.80	78.16	7.92	640	0.89/2.75/0.83
	M13	24.30	n/a	n/a	Neg	n/a
7	D11	49.50	12.82	1.8	320	0.21/0.83/1.25
	M13	31.20	n/a	n/a	Neg	n/a
8	D18	3.80	2.46	Neg	Neg	0.00/0.55/0.00
9	D6	4.70	2.68	Neg	Neg	0.00/0.00/0.00
10	D9	53.90	7.50	n/a	160	n/a
	D17	63.30	37.02	6.91	160	0.54/0.64/0.20
11	D7	3.30	1.66	n/a	Neg	n/a
	D16	10.10	1.90	Neg	Neg	0.00/0.00/0.00
12	D6	29.90	6.70	n/a	320	n/a
	D14	20.60	14.94	2.44	160	0.27/0.50/0.24
13	D33	49.60	18.43	1.2	40	0.29/0.40/0.00
14	D25	67.30	71.86	3.76	640	1.39/1.70/0.25
	M7	19.40	n/a	n/a	Neg	n/a
15	D22	40.10	24.52	2.18	80	n/a
	D26	41.10	24.00	n/a	80	n/a
16	D29	45.10	12.90	Neg	40	n/a
	D33	34.80	11.40	n/a	40	n/a
17	D21	26.50	7.28	Neg	40	0.00/0.33/0.00
18	D29	16.00	4.84	Neg	Neg	n/a
	D33	17.40	4.64	n/a	Neg	n/a
19	D21	76.60	38.32	n/a	320	n/a

	D25	71.60	35.46	3.31	320	n/a
	D33	74.40	37.32	n/a	160	0.44/0.39/0.00
	M11	31.50	n/a	n/a	Neg	n/a
20	D26	70.00	59.88	4.31	320	n/a
21	D24	62.80	62.28	5.66	160	0.38/0.44/0.12
	M11	18.20	n/a	n/a	Neg	n/a
22	D11	70.00	85.94	7.79	1280	n/a
	D15	71.70	86.88	n/a	1280	n/a
	D19	68.40	66.94	n/a	640	0.56/0.98/0.13
23	D24	51.10	67.46	7.21	640	0.75/1.18/0.00
	D28	52.00	60.10	n/a	320	n/a
	M9	50.50	n/a	n/a	80	n/a
24	D18	69.90	30.12	1.9	160	n/a
	D30	56.30	21.48	n/a	160	n/a
25	D23	42.00	39.82	2.21	320	n/a
	D30	62.00	38.70	n/a	320	0.39/0.57/0.00
	D39	60.00	32.00	n/a	160	n/a
	M8	23.60	n/a	n/a	20	n/a

Abbreviations: D, day; M, month; Neg, negative; n/a, not available.
* The ACE2-blocking titer was expressed as the reciprocal of the serum dilution giving 50% inhibition of signal compared to maximum signal. The serum with original concentration that failed to inhibit ACE2-RBD interaction was scored as negative.
# A hemagglutination titer of 1:20 or more was considered to be positive. The serum with a reciprocal titer less than 20 was considered as negative and assigned a value of 10 in the Fig. 1A.
& The frequency is defined as the percentage of spike-specific IgG (IgM or IgA) B cells in the total IgG (IgM or IgA) B cells.

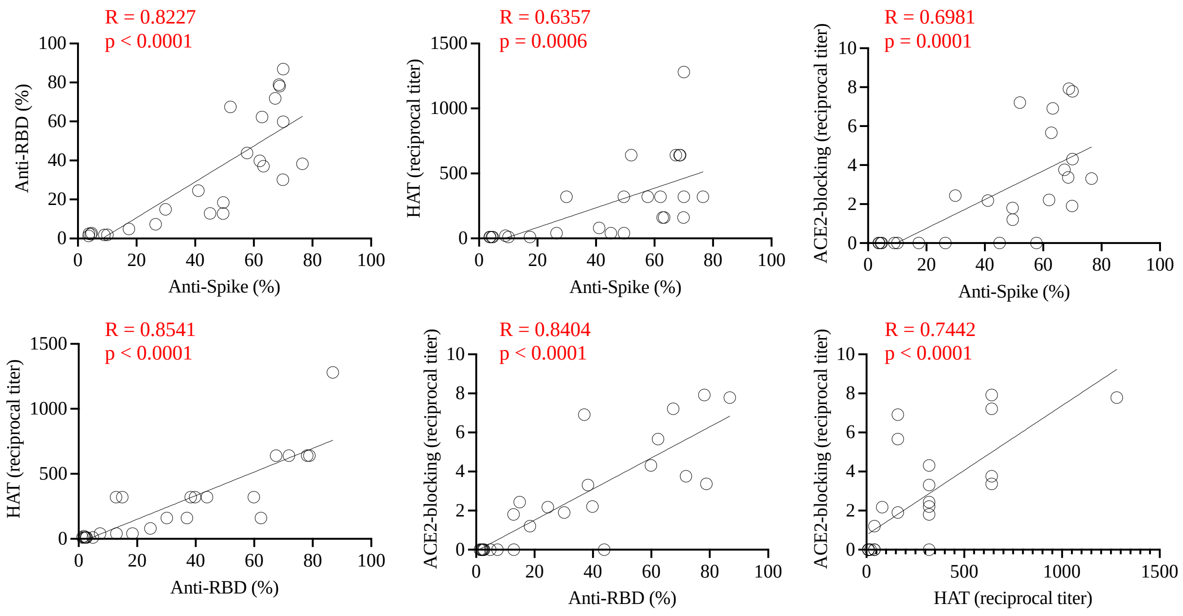

Fig. S1. Relationship of serological spike-binding, RBD-binding, hemagglutination and ACE2-blocking activity among COVID-19 patients. Linear regression is used to model the relationship between two variables.

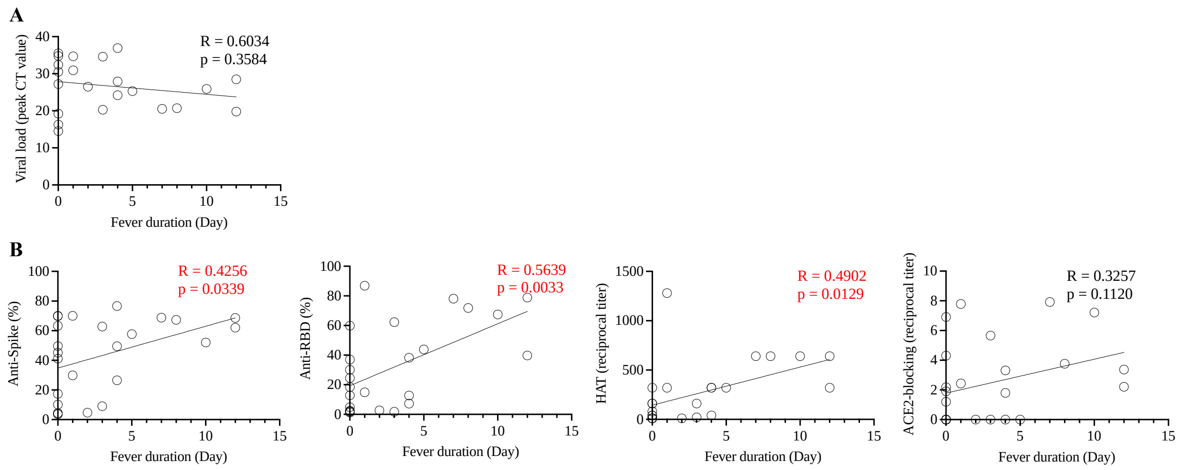

Fig. S2. (A) Relationship of fever duration and peak viral load among COVID-19 patients. (B) Relationship of fever duration and RBD-binding, hemagglutination and ACE2-blocking activity among COVID-19 patients. Linear regression was used to model the relationship between two variables.

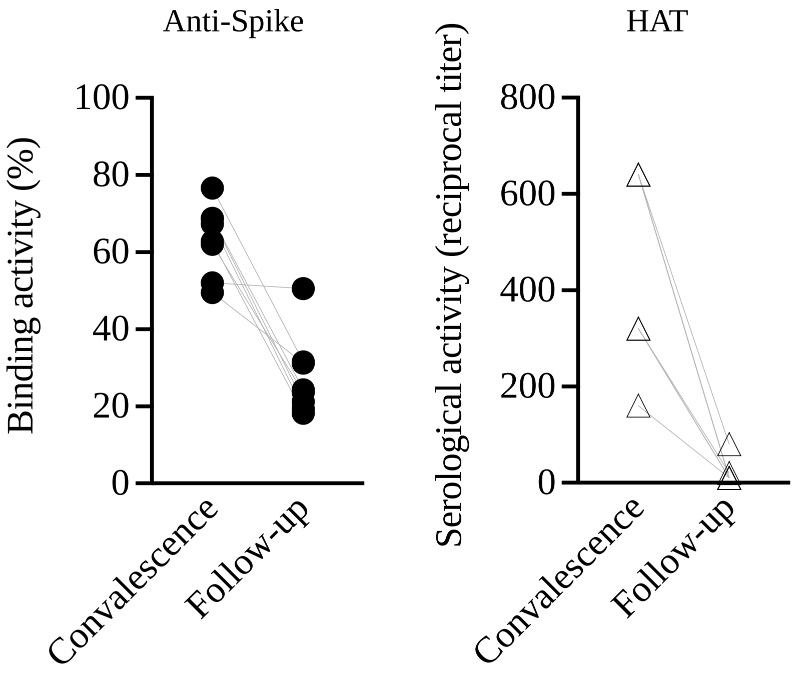

Fig. S3. Analysis of spike-binding antibody response and hemagglutination serological titer at enrolment and follow-up (n=8).

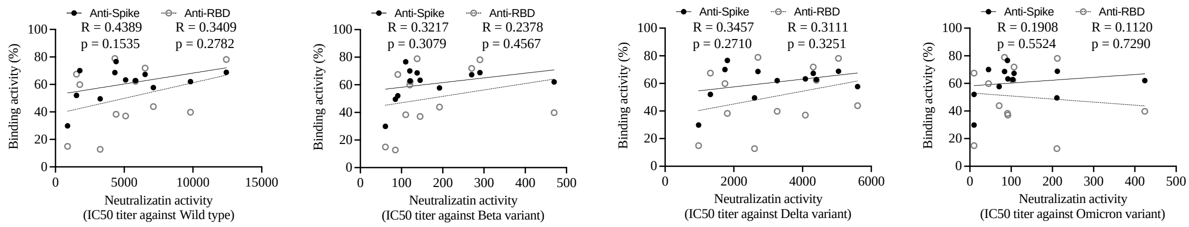

Fig. S4. Relationship of serological spike or RBD-binding activity and neutralizing activity of convalescent sera (n = 12). Linear regression is used to model the relationship between two variables.
